# Artificial intelligence in sleep diagnostics for children under 2 years of age: state of the evidence and future directions

**DOI:** 10.1183/16000617.0335-2025

**Published:** 2026-06-10

**Authors:** Molla Imaduddin Ahmed, Jessica Taytard, Stijn Verhulst, Iulia Ioan

**Affiliations:** 1 Department of Paediatric Respiratory and Sleep Medicine, University Hospitals of Leicester NHS Trust, Leicester, UK; 2 Department of Respiratory Sciences, University of Leicester, Leicester, UK; 3 Service d'Explorations Fonctionnelles Respiratoires et Somnologie, Hôpital Armand-Trousseau, Centre National de Référence des Maladies Respiratoires Rares, Paris, France; 4 Sorbonne Université, INSERM, UMR-S 1158, Paris, France; 5 Department of Paediatrics, University Hospital Antwerp, Antwerp, Belgium; 6 Université de Lorraine, CHRU-Nancy, Service d'Explorations Fonctionnelles Pédiatriques, Hôpital d'Enfants, Nancy, France; 7 Université de Lorraine, DevAH, Nancy, France

## Abstract

**Background:**

Artificial intelligence (AI) has advanced rapidly in adult sleep medicine, but its role in children under 2 years old remains unclear. This scoping review maps the current evidence on AI-enabled diagnostics in neonates, infants and toddlers, identifying challenges, opportunities and research priorities.

**Methods:**

A structured scoping review was undertaken up to April 2025, incorporating peer-reviewed studies, normative datasets, regulatory reports and evidence on consumer and commercial technologies. Studies focusing on children under 2 years were prioritised, with older cohorts reviewed where relevant to translation.

**Findings:**

No AI tool is validated for independent diagnostic use in this age group. Research prototypes, including automated polysomnography scoring, convolutional neural networks for oximetry and multimodal wearable or radar-based systems, show feasibility but remain experimental. Commercial platforms such as SleepImage, Belun Sleep Health and WatchPAT exclude children under 2 years, while consumer monitors including Owlet, Nanit and Miku are widely used but lack clinical validation.

**Conclusion:**

AI in sleep diagnostics for children under 2 years remains an emerging field with high potential but no current clinical readiness. Progress will depend on the development of infant-specific datasets, improved artefact handling, simplified monitoring strategies and human-in-the-loop workflows. Longer-term opportunities include prognostic modelling, circadian analysis and predictive closed-loop systems, but equity and regulatory clarity must guide translation to ensure safe and globally relevant impact.

## Shareable abstract

**AI in sleep diagnostics for children under 2 years is promising but unproven. Infant physiology limits current models and unregulated consumer use creates risk. Progress needs infant-specific data, robust validation and equitable global collaboration.** https://bit.ly/4u5AR8c

## Introduction

Sleep is a fundamental biological process essential for healthy development in early childhood. During the first 2 years of life, sleep supports neurocognitive maturation, emotional regulation, cardiorespiratory stability and growth. Disrupted or insufficient sleep in this period has been linked to impaired cognition, behavioural dysregulation, cardiometabolic disturbance and caregiver stress [1]. Despite this importance, sleep disorders in infants and toddlers remain underdiagnosed and poorly characterised. This reflects both the inherent challenges of assessing sleep in very young children and the lack of diagnostic frameworks tailored to their developmental stage.

Polysomnography (PSG) remains the diagnostic gold standard [2], but is technically demanding and resource-intensive to implement in neonates and infants, with specialist expertise unevenly distributed across health systems. Physiological features unique to early life, including fragmented sleep, immature respiratory control, rapidly evolving electroencephalographic (EEG) patterns and frequent arousals, complicate interpretation and limit the transferability of scoring systems derived from older children and adults [3]. Alternative approaches such as polygraphy (PG), oximetry or simplified cardiorespiratory monitoring provide partial solutions but face similar barriers. As a result, diagnosis is often delayed, inconsistent or entirely missed.

Artificial intelligence (AI) offers a potential route to address these challenges. By automating the analysis of physiological signals such as EEG, oximetry, nasal flow, ECG and cardiorespiratory patterns, AI could improve efficiency, standardise interpretation and detect clinically relevant events that are difficult for human scorers to recognise [4]. In adults, deep learning models already achieve high accuracy for sleep staging and apnoea detection, but their performance in children remains suboptimal due to developmental differences and the absence of age-specific training data [5], coupled with the high heterogeneity in aetiology of sleep disordered breathing in early childhood.

Children under 2 years therefore represent both the most challenging and the most critical frontier for AI in sleep diagnostics. Their physiology is unique, their vulnerability high and their systematic exclusion from validation pipelines problematic. Yet this same population may benefit disproportionately from innovation, as they are frequently monitored closely in both hospital and home environments, creating opportunities for novel sensing and analytic approaches.

This review maps the current evidence on AI-enabled sleep diagnostics for children under 2 years. We outline the physiological and technical challenges that complicate conventional diagnostic methods, map applications of AI across established and emerging diagnostic approaches, distinguish between research-grade, clinical and consumer-grade systems, and highlight ethical and translational issues. By clarifying current capabilities and evidence gaps, we aim to inform clinical practice and set a research agenda for this critical domain of paediatric sleep medicine.

## Methods

A structured scoping review was undertaken to identify and map the breadth of evidence relating to AI-enabled sleep diagnostics in neonates, infants and toddlers (<2 years). Searches were conducted to April 2025 across biomedical and engineering databases (PubMed/Medline, Embase, IEEE Xplore, Web of Science and Scopus), supplemented by paediatric normative datasets, regulatory listings (*e.g.* US Food and Drug Administration (FDA) 510(k) submissions, Conformité Européenne (CE)-marking databases), technical specifications and grey literature, to ensure both research prototypes and commercially marketed systems were represented. Search terms combined concepts including “artificial intelligence”, “machine learning”, “neonates”, “infants”, “sleep diagnostics”, “polysomnography”, “polygraphy”, “oximetry”, “wearable” and “contactless monitoring”, using MeSH (Medical Subject Headings), free text and Boolean operators to maximise sensitivity.

Eligible evidence applied AI, machine learning or deep learning to sleep-related signals, event detection or sleep phenotyping in children under 2 years, including but not limited to PSG, PG, oximetry, ECG, respiratory motion, audio, radar or video-derived physiological parameters. Evidence from older paediatric or adult populations was included only when there was clear methodological or translational relevance to infants and toddlers, reflecting the current structure of the field where most AI development pipelines have systematically excluded children under 2 years. Reference lists of key papers were hand-searched to identify additional relevant work.

Because the evidence base is sparse, heterogeneous and unequally distributed across modalities, systems and commercial contexts, results are organised conceptually (rather than by study design) to enable synthesis across three domains, as follows: 1) AI to enhance conventional diagnostics (PSG/PG/oximetry); 2) AI for direct diagnosis from simplified or alternative signals; and 3) AI-enabled novel monitoring or sensing platforms (wearables, radar, video, consumer devices). A formal appraisal of risk of bias or meta-analysis was not undertaken; the objective was to map the field, describe the landscape, clarify capability boundaries and identify translational gaps.

## Results

Across all sources, the evidence base for AI-enabled sleep diagnostics in children under 2 years was broad in scope but highly uneven in distribution. The overwhelming majority of published studies originated from adult or older paediatric cohorts, where physiology is more stable, scoring frameworks are well established and large annotated overnight datasets exist to support model development. In contrast, publications directly addressing neonates, infants or toddlers were uncommon, and when present were usually limited to small single-centre feasibility studies, normative data collections without diagnostic end-points or experimental sensing prototypes at an early research stage. The net pattern is that the available literature contains substantial methodological activity across multiple technical domains, but the empirical base specifically in children under 2 years remains sparse, fragmented and at an early developmental stage.

Across database searching, 534 records were identified. Following removal of 28 duplicates, 496 records were screened at title and abstract level, of which 453 were excluded, predominantly because they focused exclusively on adults or older children or because they described analytic pipelines with no paediatric applicability. 43 full texts were retrieved and assessed in detail. Eight did not meet criteria for inclusion, four because children under 2 years were not represented in the dataset (and no explicit translational relevance was articulated) and four because they did not provide any diagnostic output relevant to sleep (for example, signal quality algorithms without clinical end-points).

A further 12 records were identified from commercial, regulatory, marketing or consumer-facing sources, including FDA device clearances, CE-marked technical summaries, post-market surveillance documentation, and manufacturer white papers. Seven met eligibility criteria for descriptive synthesis. Five were excluded, three because they comprised promotional descriptions of products without technical content and two because they lacked sufficient detail to determine whether AI was genuinely used to support classification, staging or prediction. In total, 42 sources were therefore included in the qualitative synthesis.

Taken together, the included evidence reflects four broad patterns. First, infant-specific research exists but is small, early-phase, exploratory and generally conducted in single specialist centres. Second, the majority of AI development has taken place in adults and older children, with “future paediatric applicability” often stated, but without infant validation. Third, clinically regulated AI-enabled auto-scoring platforms do exist, but they explicitly exclude children under 2 years. Fourth, the only AI technologies currently used at scale in neonates and infants are consumer baby monitors that operate outside medical regulatory frameworks and lack validation against PSG. The overall conclusion from this evidence landscape is that feasibility signals are present across multiple modalities, but the entire evidence base in children under 2 years remains pre-clinical. No system has undergone rigorous prospective validation against PSG in this age group and there is no evidence of generalisability across different disease aetiologies, physiological phenotypes, gestational ages or developmental stages. All current AI applications in infants should therefore be regarded as exploratory rather than diagnostic.

### AI to enhance conventional diagnostics

PSG, PG and oximetry remain the foundations of paediatric sleep diagnostics, yet their application in children under 2 years is fraught with challenges, including poor tolerance, signal instability and the incompatibility of infant physiology with scoring rules derived from older populations. AI has therefore been explored primarily as a tool to enhance these established modalities rather than replace them, with the goals of automating labour-intensive scoring, reducing inter-scorer variability and extracting greater value from existing signals [6].

Automated sleep staging has been the earliest domain of work. Machine learning approaches were first applied to infant EEG and cardiorespiratory patterns in small research cohorts, including studies motivated by sudden infant death syndrome, but these never reached clinical translation due to limited datasets and inconsistent accuracy [7]. More recent deep learning systems have achieved substantially improved performance. U-Sleep, for example, achieved high concordance with expert scorers in adolescents but performed markedly less well in children under 2 years, likely reflecting unstable EEG patterns and fragmented sleep architecture in infancy [8]. Newer models such as PFTSleep, a transformer-based system analysing entire nights of PSG [9], and PedSleepMAE, which employs generative multimodal modelling to detect apnoeas, hypopnoeas and arousals [10], illustrate current methodological innovation but remain entirely unvalidated in infants and toddlers. AI applied to PG and oximetry shows similar patterns. Deep learning applied to nocturnal peripheral oxygen saturation (*S*_pO_2__) has improved discrimination of sleep apnoea severity in older children [11, 12] and PG has large normative datasets available in infancy [13, 14], but no AI model has been trained or validated in infants using these data.

Commercial auto-scoring platforms further demonstrate the current state of clinical maturity. EnsoSleep (EnsoData, USA) pre-scores PSG and home studies and has FDA clearance for patients aged ≥13 years [15]. Somnolyzer (Philips Respironics) reduces technologist scoring workload [16], while DOMINO AI (SOMNOmedics) provides CE-marked pre-classification of sleep stages and respiratory events [17]. All three exclude children under 2 years.

### AI for direct diagnosis of sleep disorders

Beyond augmenting conventional diagnostics, a parallel body of work has sought to determine whether AI can directly diagnose sleep disorders from simplified or alternative signals. For children under 2 years, this remains largely experimental, but several proof-of-concept studies illustrate feasibility.

Some of the most clinically relevant examples relate to apnoea of prematurity. Zuzarte *et al*. [18] showed that algorithms trained on vital signs could predict apnoeas seconds before clinical recognition. Shirwaikar *et al*. [19] applied artificial neural networks, support vector machines and k-nearest neighbour classifiers, demonstrating feasibility in predicting apnoeas in preterm neonates. Cohen and de Chazal [20] integrated ECG and *S*_pO_2__ in 396 infants to detect apnoeas with high sensitivity and specificity, and Varisco *et al*. [21] confirmed that machine learning could reliably identify central apnoea in preterm neonates. These examples collectively represent clear demonstrations of feasibility in this age group.

Approaches that rely on less intrusive sensor configurations have also been explored. The LittleBeats™ infant-wearable platform integrates ECG, accelerometry and audio, and applies transformer models to classify sleep–wake states and apnoea-related events, demonstrating data quality broadly comparable to gold-standard devices [22]. Contactless models have achieved significant technical advances in older cohorts. Wang *et al*. [23] applied millimetre-wave radar combined with oximetry and deep learning to detect obstructive sleep apnoea in children, though infants were excluded. Camera-based photoplethysmography (PPG) and thermal imaging have been tested in neonates to estimate respiratory signals and detect apnoeas [24].

### Novel AI-based monitoring and sensing

AI is also being applied to novel monitoring and sensing technologies designed to capture physiological or behavioural sleep data in less intrusive ways than PSG, an approach well suited to early childhood. Research-grade radar-based systems have demonstrated continuous respiratory monitoring without cannulae or electrodes. Beltrão *et al*. [25] demonstrated the feasibility of Doppler radar in preterm neonates, extracting continuous cardiorespiratory signals and Wang *et al*. [23] combined millimetre-wave radar with oximetry and deep learning to detect obstructive sleep apnoea in children. Video-based sensing is similarly active. Villarroel *et al*. [26] applied camera PPG to preterm infants to measure heart and respiratory rates. Lorato *et al*. [27] combined thermal and RGB video to estimate respiration, and other groups have trained algorithms to detect apnoeas from neonatal video recordings [28]. These studies collectively demonstrate that clinically relevant signals can be extracted without physical contact, but robust validation against PSG in children under 2 years is still lacking.

Consumer devices provide another major source of innovation and data generation. The Owlet Smart Sock uses PPG to track oxygen saturation and heart rate [29, 30]. The CuboAi Sleep Sensor Pad detects breathing motion under the mattress [31]. Video-based platforms such as Nanit and Miku Pro combine cameras with motion or radar analytics to infer sleep patterns, to generate sleep–wake reports and behavioural coaching [32, 33]. The Snuza Hero/Pico provides wearable abdominal sensors for motion-based breathing detection [34]. Empatica devices offer multimodal physiological tracking, including heart rate and electrodermal activity, and may be useful in paediatric sleep and seizure detection [35]. The Withings Sleep Mat employs under-mattress sensors to monitor respiration and sleep patterns and could also have relevance for paediatric and infant sleep and apnoea monitoring; however, it is currently recommended only for adults and has not yet been validated in children [36]. None have been validated against PSG in children under 2 years.

Several “medical-grade” AI-enabled platforms occupy an intermediate position. The Belun Ring, based on PPG and accelerometry, has been evaluated in children aged 5–18 years but showed only moderate correlation with PSG and wide discrepancies in apnoea–hypopnoea indices [37]. SleepImage, which applies cardiopulmonary coupling analysis to ECG or PPG signals, is FDA-cleared for children aged ≥2 years [38]. WatchPAT, based on peripheral arterial tonometry, is validated only from adolescence [39]. These examples highlight the systematic exclusion of neonates and infants from clinical validation despite the fact that the underlying sensing hardware is often technically capable of acquiring usable data in early life. Table 1 summarises the main research, commercial and consumer technologies relevant to children under 2 years.

**TABLE 1:** Summary of artificial intelligence (AI)-enabled sleep technologies relevant to children under 2 years

Device/platform	Signal type	AI functionality	Age range studied	Validation/regulatory status
**Research-only systems**				
U-Sleep [8]	PSG (EEG, respiratory signals)	Automated sleep staging	Children/adolescents	Poorer accuracy in <2 years; not validated for infants
PFTSleep [9]	PSG (full-night transformer)	Contextual sleep staging and apnoea detection	Adults	Research only; no infant validation
PedSleepMAE [10]	Multimodal PSG	Generative multimodal detection of events	Children/adolescents	Research only
CNNs for infant oximetry [12, 13]	*S*_pO_2__	Burden analysis, event detection	Preterm, term infants	Early feasibility; no regulatory status
Generative AI [40]	PSG clinical notes	Extracts structured sleep parameters	Adults	Research only
Radar/video prototypes [25–28]	Radar, thermal, RGB video	Contactless respiratory/sleep monitoring	Preterm/infants (small studies)	Feasibility only
LittleBeats [22]	ECG, accelerometry, audio	Transformer for sleep/wake and apnoea	28 infants	Research prototype
Smartphone acoustic AI [41, 42]	Snoring/breathing sounds	OSA screening	Adults	Research only
**Commercial medical-grade**				
SleepImage [38]	ECG (CPC analysis)	Sleep quality and OSA indices	≥2 years (off-label <2)	FDA-cleared ≥2 years; not validated in infants
Belun Ring [37]	PPG+accelerometer	Sleep staging, AHI estimation	≥5 years	CE-marked; excluded <5 years
WatchPAT [39]	PAT+actigraphy+*S*_pO_2__	OSA diagnosis	≥12 years and adults	FDA-cleared ≥12 years
Empatica [35]	EDA, HR, motion	Event/autonomic monitoring	Adults, older children	Research use only in paediatrics
**Consumer-facing devices**				
Owlet Smart Sock/ Dream Sock [30]	PPG	HR and *S*_pO_2__ alerts	0–18 months	Rebranded as “wellness device”; not FDA-cleared for diagnostics
CuboAi Sleep Pad [31]	Motion (under-mattress)	Breathing movement alerts	Infants	Consumer only; not validated
Nanit+Breathing Wear [32]	Video+pattern recognition	Sleep tracking, respiration inference	0–24 months	Consumer only
Miku Pro [33]	Radar+video	Contactless respiration and sleep tracking	Infants	Consumer only
Snuza Hero/Pico [34]	Abdominal motion	Breathing motion alerts	Neonates	Consumer only
Withings Sleep Mat [35]	Under-mattress sensors	Sleep staging, HR, movement	Adults	Not validated in infants/children

AHI: apnoea–hypopnoea index; CE: Conformité Européenne; CNN: convolutional neural network; CPC: cardiopulmonary coupling; ECG: electrocardiogram; EDA: electrodermal activity; EEG: electroencephalogram; FDA: Food and Drug Administration; HR: heart rate; OSA: obstructive sleep apnoea; PAT: peripheral arterial tone; PPG: photoplethysmography; PSG: polysomnography; RGB: red, green, blue; *S*_pO_2__: peripheral oxygen saturation.

## Discussion

A central pattern emerging from this review is that infants under 2 years are markedly under-represented in AI development and validation for sleep diagnostics, despite being the age group in which conventional testing is most difficult and where clinical need is considerable. Although AI has achieved state-of-the-art performance in adult sleep medicine and is increasingly adopted in older paediatric populations, translation into the first 2 years of life remains extremely limited. This is not a trivial developmental gap that could be resolved by adding more data from older cohorts or by minor retraining of existing models. Rather, it reflects a fundamental mismatch between the physiology of early life and the assumptions embedded within current AI pipelines.

The diagnostic evaluation of sleep in children under 2 years is governed by biological and methodological factors that differ qualitatively from older children and adults. Neonates and infants breathe more rapidly and variably, with frequent periodic breathing and immature central control. Paradoxical chest and abdominal movements are common in healthy infants, limiting their reliability as markers of obstruction [43]. Sleep itself is highly fragmented, with frequent arousals and awakenings for feeding, which reduces sleep efficiency and compromises the acquisition of stable epochs suitable for conventional scoring. Sleep architecture also evolves rapidly across the first 2 years of life. Rapid eye movement (REM) sleep proportion is highest in infancy, EEG signatures undergo continuous maturation and cortical rhythms lack the temporal stability seen in older children. Accurate scoring therefore requires specialised expertise and reference to the American Academy of Sleep Medicine infant rules, yet inter-scorer variability remains substantial [44]. Normative data illustrate this physiological volatility. Brockmann *et al*. [45] reported high central apnoea indices at 1 and 3 months of age that declined with maturation, Vézina *et al*. [46] documented marked variability in apnoea indices among more than 500 1-year-olds and Singh *et al*. [47] showed that daytime PSG yields comparable diagnostic information to overnight studies in infants ≤2 months. Technical factors further amplify these challenges. Small tidal volumes reduce the accuracy of nasal pressure and thermistor signals, and cannula displacement is frequent. End-tidal CO_2_ monitoring is often unreliable in neonates due to absent waveform plateaus, necessitating transcutaneous CO_2_ monitoring, which itself requires calibration and may cause skin irritation [48]. Portable PG has been applied successfully in some young children, but infant studies consistently report high artefact rates and variable reproducibility [49]. Resource intensity adds further constraints, as infant PSG often requires prolonged recordings, scoring is time-consuming due to frequent arousals and atypical events, and diagnostic reliability is undermined by high inter-scorer variability even in specialist centres [50].

Table 2 outlines the principal conventional diagnostic approaches in infants, their technical limitations, the current state of AI evaluation and the absence of validated applications in children under 2 years.

**TABLE 2:** Conventional diagnostic modalities in children under 2 years: technical limitations, artificial intelligence (AI) evaluation and validation status

Modality	Key technical/artefact challenges in <2 years	AI evaluation status	Validation status in <2 years
**Polysomnography**	Highly fragmented sleep with frequent arousals compromising stable scoring epochs; high inter-scorer variability; technically demanding and resource-intensive [44, 50]	Automated sleep staging and event detection models well developed in adults and older children [8–10]	No AI tool validated for independent diagnostic use
**Polygraphy**	Small tidal volumes reduce nasal pressure and thermistor accuracy; cannula displacement frequent; high artefact rates; variable reproducibility; paradoxical breathing common in healthy infants [43, 49]	AI methods largely developed in older paediatric or adult cohorts [11–14]	No validated AI models in <2 years
**Nocturnal oximetry**	Artefact common in infant monitoring; developmental variability in central apnoea indices complicates interpretation [13, 45]	Deep learning approaches applied to *S*_pO_2__ primarily in older children [11, 12]	No regulatory-cleared AI applications in infants
**Capnography (end-tidal/transcutaneous CO_2_)**	End-tidal CO_2_ often unreliable in neonates due to absent waveform plateaus; transcutaneous CO_2_ monitoring requires calibration and may cause skin irritation [48]	No validated AI applications described in children or infants	No validated AI applications in <2 years

*S*_pO_2__: peripheral oxygen saturation.

These combined physiological and technical factors explain why diagnostic thresholds derived from older populations cannot simply be applied to children under 2 years and why AI algorithms trained on adult or school-aged datasets perform poorly. Sleep in the first 2 years of life is not a simplified or “smaller” version of older child sleep. It is categorically distinct. EEG signatures undergo rapid transitions, circadian structures are immature, arousal thresholds are unstable, ventilatory control systems are still consolidating and movement artefact is pervasive. These developmental properties disrupt the statistical stationarity and temporal regularities that underpin successful model performance in adolescents and adults; systems optimised for older populations do not simply “scale down” into infancy.

Table 3 illustrates that infant sleep physiology is developmentally dynamic rather than stable, challenging the statistical assumptions of most current AI pipelines that rely on stationary signals and fixed diagnostic thresholds.

**TABLE 3:** Developmental changes in sleep physiology across the first 2 years of life and implications for artificial intelligence (AI) modelling

Domain	Neonates	Infants (<2 years beyond neonatal period)	Implications for AI systems
**Sleep architecture [44]**	Active *versus* quiet sleep; poorly differentiated states	REM/NREM differentiation emerging but unstable; persistent fragmentation	Unstable stage definitions limit application of adult-derived scoring models
**REM proportion [44]**	High	Gradual reduction but remains higher than in older children	Different stage distribution reduces transferability of adult-trained models
**Sleep fragmentation [44, 50]**	Frequent awakenings; short sleep cycles	Fragmentation common; consolidation gradually improves	Frequent arousals reduce availability of stable epochs for automated scoring
**EEG patterns [3, 44]**	Discontinuous and immature EEG background patterns	Rapid maturation; evolving spindles and slow-wave activity	Immature and evolving EEG signatures limit transferability of adult-trained staging algorithms
**Respiratory control [43, 45]**	Immature central control; periodic breathing common	Central events decline but remain developmentally variable	Physiological central events may be misclassified as pathological
**Apnoea indices [45, 46]**	High central apnoea indices common in health	Wide normative variability	Fixed diagnostic thresholds derived from older populations may be inappropriate
**Thoracoabdominal movement [43]**	Paradoxical breathing common in health	Coordination improves but remains variable	Paradoxical motion may be misinterpreted as obstruction
**CO_2_ monitoring reliability [48]**	End-tidal CO_2_ often unreliable; transcutaneous required	Signal quality improves but remains technically challenging	Hypoventilation assessment limited by signal instability
**Movement artefact [49]**	High	High	Frequent artefact complicates automated signal interpretation

EEG: electroencephalogram; NREM: nonrapid eye movement; REM: rapid eye movement.

Against this physiological backdrop, the pattern across established PSG/PG/oximetry AI becomes clearer. AI-enhanced scoring pipelines are now technically strong in older populations, and in some cases are fully operationalised clinically. However, these systems have not been validated in infants not because of lack of interest, but because the underlying signal distributions are fundamentally different. A deep learning model that “sees” stable sleep stages, stable respiratory patterns and stable EEG in a 15-year-old has never been exposed to the volatility, nonlinearity and discontinuity that define infant sleep. The fact that commercial auto-scoring platforms explicitly exclude this age group is therefore less an omission and more a tacit acknowledgement that the analytic primitives they rely upon do not exist reliably before 2 years of age.

Parallel efforts that attempt to bypass PSG entirely and infer diagnostic states directly from simplified or alternative signals show a different constraint. The feasibility signals around apnoea prediction in preterm neonates are compelling and demonstrate that clinically meaningful information is present in the data streams already routinely captured in neonatal and paediatric practice. But the scope remains narrow, focused almost entirely on apnoea, and studies are small, single-centre and without external validation. These approaches do not yet address the harder questions that actually drive diagnostic uncertainty in infants, namely arousal burden, hypoventilation, autonomic instability, sleep fragmentation phenotypes or developmental transitions in sleep architecture. Evidence of possibility exists, but evidence of breadth, scalability or clinical substitutability does not.

The newest sensing paradigms reveal a third tension. Contactless radar and video systems are inherently aligned with infant tolerability constraints because they bypass the core feasibility limitations of PSG in early life. Yet their evaluation remains largely confined to prototype-stage research without PSG-calibrated validation. Meanwhile, the only devices in this sensing category used at population scale are consumer monitors with no clinical regulatory clearance and no evidence of PSG-equivalent performance. In effect, the most physiologically suited approaches are the ones least formally validated and the most heavily regulated systems explicitly exclude the age group where the marginal diagnostic value could be greatest.

The integration of AI into sleep diagnostics for children under 2 years presents a complex set of ethical, regulatory and equity challenges. This age group is paradoxically both the most excluded from clinical validation and the most targeted by consumer-facing technologies. One prominent issue is the blurred line between consumer and clinical products. Devices such as the Owlet Dream Sock, Nanit and Miku Pro are marketed directly to parents of neonates and infants [30, 32, 33]. They are classified as “wellness” monitors rather than medical devices, which exempts them from formal regulatory pathways such as FDA clearance, despite advertising features that include alerts for changes in sleep or breathing. In contrast, regulated clinical platforms such as EnsoSleep or the Belun sleep health are subject to oversight but are not validated for children under 2 years [37, 38]. The result is a regulatory paradox in which the youngest and most vulnerable patients are largely exposed to unregulated consumer devices, while being excluded from validated clinical tools.

Privacy and data security are also critical concerns, particularly for video- and audio-based monitors. These systems often rely on continuous recording in the home, with data transmitted to cloud servers. Policies around storage, retention and secondary use are not always transparent and infants cannot themselves consent to long-term biometric data collection. Although frameworks such as GDPR (General Data Protection Regulation) offer protection, they may not fully address the unique challenges of paediatric data governance.

Bias further complicates adoption. Most AI systems are trained on data from adults or school-aged children in high-resource settings, meaning they generalise poorly to neonates and toddlers. Under-representation of diverse ethnic, socioeconomic and geographic groups risks exacerbating inequities in paediatric sleep medicine rather than reducing them.

These issues intersect with questions of explainability, accountability and clinical oversight. Algorithms intended for infants must provide interpretable outputs and uncertainty estimates, enabling clinicians to adjudicate ambiguous results. Even validated systems require post-market surveillance to ensure safety, as models may drift when applied to new populations or following software updates. Yet accountability for failures is diffuse; consumer manufacturers often disclaim medical responsibility, while clinical users may be uncertain about liability in cases of misdiagnosis.

Finally, global equity remains an essential consideration. AI could widen access to sleep diagnostics in settings where PSG is unavailable, but only if datasets are diverse and representative. Without deliberate inclusion, tools may be optimised for wealthy populations while performing less effectively elsewhere.

For AI to be responsibly integrated into sleep diagnostics for infants and toddlers, development must be anchored in age-specific datasets, transparent data governance, rigorous human oversight, accountable regulatory pathways and intentional strategies to avoid widening existing inequities. What is required is not a marginal adaptation of adult-grade AI, but an explicit re-engineering of the diagnostic paradigm for early childhood. This includes building sufficiently large and diverse infant cohorts that capture true developmental heterogeneity, developing signal processing pipelines optimised for the artefact profiles of early life and evaluating performance across multiple disease states and demographic contexts. Without these structural shifts, AI will continue to progress in the populations where the physiological problem is simplest and the regulatory risk lowest, while remaining absent where the unmet clinical need and potential lifetime impact are greatest.

### Future directions

Current evidence confirms that no AI system is validated for independent diagnostic use in children under 2 years. Nonetheless, several scientific and translational pathways offer promise, provided they recognise the distinctive physiology and clinical needs of neonates, infants and toddlers.

The foremost priority is the creation of infant-specific datasets. Existing models underperform because they are trained almost entirely on adult or school-age cohorts, systematically excluding younger children. Large, multicentre collaborations are needed to capture the diversity of early childhood physiology, including healthy infants, preterm neonates and those with disease. Federated learning and international data-sharing could help overcome privacy and resource barriers.

Signal quality control is another urgent target. Infant PSG and PG are especially prone to artefact from movement or poor sensor tolerance. AI tools that assess signal quality, denoise recordings or reconstruct corrupted data, already used in adult PSG, could improve reliability and reduce technologist burden.

Beyond event detection, AI may offer richer insights into developmental physiology. Monitoring circadian rhythm consolidation through actigraphy, video or multimodal signals could help track neurodevelopmental trajectories. Digital phenotyping, achieved by integrating multimodal data such as EEG, oximetry and behavioural cues, may allow algorithms to identify infant subgroups with distinct risk profiles and inform personalised management strategies. Prognostic modelling could then stratify risk further, distinguishing those with transient central apnoea from those likely to develop persistent sleep-disordered breathing or chronic lung disease.

In management, AI-driven CPAP devices and predictive models show promise for optimising adherence and personalising therapy, while mobile health apps and wearables may extend access to home-based obstructive sleep apnoea detection [51]. More broadly, AI now achieves accuracy in automated PSG scoring comparable to human scorers and machine learning approaches support risk stratification, disorder endotyping and prediction of treatment response [52, 53]. These advances open pathways toward precision and personalised sleep medicine, but progress remains constrained by the absence of representative paediatric datasets, particularly in infants. Recent work illustrates how generative AI can accurately extract core sleep parameters from unstructured PSG reports, achieving performance comparable to human annotation and even improvements in extracting sleep onset latency [40]. Applied to neonatal and infant sleep medicine, such methods could enable large-scale data mining of clinical records, accelerating the development of infant-specific datasets that are urgently needed. However, real-world implementation also faces persistent barriers, including lack of transparency, limited clinician training, ethical and regulatory uncertainties, and concerns about bias and patient safety [54]. Addressing these challenges will be essential to ensure safe and equitable translation into infant care.

Wearables and textiles also represent an important future direction. Advances in smart textiles and wireless systems support continuous, noninvasive monitoring at home, offering alternatives where PSG is impractical [55]. Pressure-sensing mats and textile platforms show potential for scalable monitoring of posture, respiration and cardiovascular signals, though most remain in early stages [56]. Intelligent sock-based systems integrating oximetry, heart rate and temperature monitoring with mobile app alerts have also demonstrated feasibility [57]. In parallel, AI has shown strong performance in diagnosing sleep apnoea, classifying disorder subtypes and predicting treatment outcomes [51, 58]. The convergence of these trends highlights an opportunity to develop infant-specific AI applications that integrate multimodal wearable and textile data for safer monitoring, better diagnostic capability and personalised care.

Practical deployment will depend on workflow integration. Human-in-the-loop models, where AI pre-scores or flags uncertain epochs, are already common in adult practice and may represent the safest pathway for infants [59]. Importantly, these systems must provide uncertainty estimates and interpretable outputs to preserve clinician oversight. Looking further ahead, predictive and closed-loop systems, capable of anticipating apnoeas or adjusting respiratory support, offer a transformative but high-stakes frontier.

Finally, progress must be equitable. If trained only on high-resource populations, infant AI will fail to generalise and risks worsening global disparities. Deliberate inclusion of diverse datasets and international collaboration will be essential to ensure fairness and clinical utility worldwide. Systematic reviews confirm that AI achieves high diagnostic accuracy for OSA across multiple signals, often comparable or superior to conventional methods, but most studies remain heterogeneous and adult-focused, underlining the urgent need for large, standardised infant datasets [60].

At present, AI in sleep diagnostics for children under 2 years remains an emerging field defined more by potential than by reality. The pathways outlined here illustrate how feasibility work could evolve into safe, equitable and clinically meaningful adoption.

## Conclusion

AI in sleep diagnostics for children under 2 years remains promising but fundamentally unproven. Infants have highly variable sleep–wake patterns, immature respiratory control and rapidly evolving physiology; factors that undermine algorithms trained on older cohorts and explain their routine exclusion from clinical validation. Although early research prototypes show feasibility, evidence is limited and far from deployment-ready. Current commercial medical-grade platforms are approved only for older children or adults, while consumer monitors, despite widespread use in infancy, lack regulatory clearance. This creates a clear paradox: the youngest and most vulnerable children are the least represented in validation and yet the most exposed to unregulated technologies.

Closing this gap will require large, representative infant datasets, improved signal quality and artefact handling, and rigorous benchmarking against established reference standards. Progress will depend on transparent, human-in-the-loop workflows and clear reporting of uncertainty. Equally, innovation must prioritise equity and global collaboration to prevent widening disparities in access and representation.

At present, AI in sleep diagnostics for children under 2 years remains at the exploratory stage. With focused development, robust validation and responsible regulation, it holds the potential to transform a labour-intensive, resource-limited process into a safe, scalable and clinically meaningful tool for paediatric sleep medicine. The iDREAM ERS Clinical Research Collaboration on sleep-disordered breathing in children under 2 years aims to offer a structured platform for harmonised protocols, shared datasets and coordinated multi-centre studies, which is an essential step toward generating the robust evidence base needed to advance infant sleep diagnostics and to support the safe, equitable adoption of AI across diverse populations.

**Questions for future research**

- What reference standards are appropriate for training and validating AI models in infant sleep diagnostics, given the limitations of adult-derived scoring rules?
- Can AI reliably distinguish pathological sleep-disordered breathing from normal developmental phenomena such as periodic breathing and transient central apnoea?
- What is the minimum physiologically sufficient sensor configuration, wearable or contactless, that can reliably detect clinically meaningful sleep-related respiratory and arousal events in infants, when benchmarked against established reference standards?
- Does AI-assisted sleep analysis improve clinically meaningful outcomes in infant services, including time to diagnosis, treatment decisions and caregiver burden?
- What is the safest and most effective role for human–AI collaboration in infant sleep medicine, particularly with respect to uncertainty, explainability and clinical oversight?
- How can wearable and home-monitoring technologies be integrated into infant care without increasing harm, bias or health inequity?
